# Prognostic Value of Des-Gamma-Carboxy Prothrombin in AFP-Negative Hepatocellular Carcinoma Patients Following Liver Resection: *A Multicenter Study*

**DOI:** 10.7150/jca.112394

**Published:** 2025-06-12

**Authors:** Si-Yu Liu, Mu-Gen Dai, Wen-Feng Lu, Lei Liang, Jun-Wei Liu, Zhuo-Kai Li, Bin Ye

**Affiliations:** 1 School of Pharmaceutical Sciences, Zhejiang Chinese Medical University, Hangzhou, Zhejiang Province, China.; 2 Department of Laboratory Medicine, The Fifth Affiliated Hospital of Wenzhou Medical University, Zhejiang Province, China.; 3 Department of Gastroenterology, The Fifth Affiliated Hospital of Wenzhou Medical University, Zhejiang Province, China.; 4 Department of Hepatobiliary Surgery, Eastern Hepatobiliary Surgery Hospital, Second Military Medical University (Navy Medical University), Shanghai, China.; 5 General Surgery, Cancer Center, Department of Hepatobiliary & Pancreatic Surgery and Minimally Invasive Surgery, Zhejiang Provincial People's Hospital, Affiliated People's Hospital, Hangzhou Medical College, Hangzhou, Zhejiang, China.; 6 Department of Hepatobiliary Surgery, The Fifth Affiliated Hospital of Wenzhou Medical University, Zhejiang Province, China.

**Keywords:** hepatectomy, des-gamma-carboxy prothrombin, recurrence, survival, hepatocellular carcinoma

## Abstract

**Background:** Hepatocellular carcinoma (HCC) is a major global health concern with high postoperative recurrence rates. Des-gamma-carboxy prothrombin (DCP) is a promising biomarker for HCC prognosis, but its optimal cutoff value remains unclear, especially in AFP-negative patients. This study aimed to determine the ideal cutoff value of DCP in AFP-negative HCC patients following liver resection and to investigate its impact on long-term outcomes.

**Methods:** This multicenter retrospective study included 661 patients who underwent curative HCC resection between 2015 and 2020 at three Chinese hospitals. Patients with AFP levels < 20 ng/mL were included. The primary endpoints were overall survival (OS) and time to recurrence (TTR). DCP levels were categorized as low (≤ 600 mAU/ml) and high (> 600 mAU/ml).

**Results:** Among the 661 patients (median age 56 years; 88.4% men), 477 had low DCP levels and 184 had high DCP levels. Patients with high DCP levels had more aggressive tumor characteristics, including larger tumor size, microvascular invasion, and macrovascular invasion. The 5-year OS rates were 76.3% in the low DCP group vs. 57.6% in the high DCP group (*P* < 0.001), and the 5-year recurrence rates were 44.9% vs. 61.0% (*P* < 0.001), respectively. Multivariable analysis showed that high DCP levels were an independent risk factor for decreased OS (HR 1.548, 95%CI 1.135-2.111; *P* = 0.006) and increased TTR (HR 1.390, 95%CI 1.081-1.787; *P* = 0.010).

**Conclusion:** A DCP cutoff value of 600 mAU/ml effectively stratifies AFP-negative HCC patients into high- and low-risk groups for survival and recurrence after liver resection. This cutoff value can guide clinical decision-making and improve prognostic accuracy.

## Introduction

Hepatocellular carcinoma (HCC), a type of liver cancer, ranks as the sixth most prevalent cancer and the third major contributor to cancer-related deaths globally^1^. In general, 40% of HCC occur in China, with approximately 85% of these cases being associated with hepatitis B virus (HBV)^2^,^3^. Surgical resection treatment is recognized as the preferred method for HCC patients who conform to the hepatectomy indications^4^,^5^. Unfortunately, long-term survival after liver resection for HCC remains less impressive, compromised by high rates of postoperative recurrence, which can range from 50% to 70% within 5 years after surgery. Therefore, a further recognition of tumor characteristics and recurrence-related risk factors for HCC patients are indispensable.

Traditional HCC staging, such as Barcelona clinic liver cancer (BCLC) stage and American Joint Committee on Cancer (AJCC) TNM stage, and serum alpha-fetoprotein (AFP) are currently being widely used to guide clinical practice^6^,^7^. AFP, a frequently employed biomarker for HCC, has a crucial role in the monitoring, diagnosis, and prognostic assessment of HCC patients. It is important to note that AFP, when used at a threshold level of 20 ng/mL, has limited efficacy in detecting HCC. Several studies have demonstrated that the diagnostic sensitivity and specificity of AFP to detect HCC are approximately 60% and 80%, respectively^8^. This means that a proportion of HCC patients have negative AFP levels at initial diagnosis, especially in patients with small HCC^9^. Furthermore, the global incidence of AFP-negative HCC is on the rise, especially among those of non-viral etiology^10^.

Des-gamma-carboxy prothrombin (DCP), also known as prothrombin induced by vitamin K absence or antagonist-II (PIVKA-II), has been found to be another useful tumor marker for HCC^11^. Previous studies have shown that DCP is a more effective biomarker for the diagnosis of HCC compared to AFP. Although, DCP, using various cutoff values according to different studies, is also an independent risk factor for HCC patients who underwent curative resection, there is no widely accepted optimal cutoff value for DCP so far^12^-^15^. Many studies did not consider the effect of AFP when determining the cutoff value of DCP. DCP and AFP reportedly demonstrate that consensus appears to have been reached on them being independent HCC markers^16^,^17^. However, when selecting the optimal cutoff value of DCP, AFP and DCP will inevitably produce the so-called superimposed effect on the prognosis. For instance, when HCC patients had a very high AFP level but normal DCP levels, their long-term prognosis were poor, which may be influenced by the high AFP levels. As such, using a multicenter database from China, the aim of this study was to identify ideal cutoff value of DCP in AFP-negative HCC patients following liver resection and investigate clinical features, long-term oncologic prognosis of patients with low or high DCP levels.

## Methods

### Study design and patient cohort

Patients who underwent curative HCC resection between 2015 and 2020 were identified from databases at three Chinese hospitals: the Fifth Affiliated Hospital of Wenzhou Medical University, Eastern Hepatobiliary Surgery Hospital, Zhejiang Provincial People's Hospital. Postoperative pathological examination confirmed the presence of HCC. Curative liver resection was defined as the complete removal of HCC with a microscopically negative margin, also known as R0 resection.

The inclusion criteria were: 1) R0 resection; 2) no history of previous anti-cancer therapy; 3) no history of other malignancies; 4) no warfarin intake before surgery; and 5) no obstructive jaundice. Exclusion criteria were as follows: 1) AFP positive; 2) Eastern Cooperative Oncology Group (ECOG) status score more than 2; 3) under 18 years old; 4) 90-day postoperative mortality; 5) presence of tumor rupture; and 6) incomplete preoperative or postoperative follow-up medical record. The study was conducted in accordance with the Declaration of Helsinki and the Ethical Guidelines for Clinical Studies and was approved by the Institutional Review Boards at the participating hospitals.

### Data collection and variable definitions

Risk factors of survival and recurrence were assessed in relation to the patient conditions, tumor characteristics, and treatment methods. The demographic and clinical laboratory test results included the age, gender, American Society of Anesthesiologists (ASA) score, body mass index (BMI), hepatitis B virus (HBV), hepatitis C virus (HCV), cirrhosis, portal hypertension, Child-Pugh grade, preoperative serum alanine aminotransferase (ALT), aspartate aminotransferase (AST). Portal hypertension was defined as the condition characterized by the presence of esophageal varices or splenomegaly accompanied by a platelet count ≤ 100×10^9^/L. The tumor-related variables encompassed the maximum tumor diameter, tumor number (solitary or multiple), presence of microvascular invasion (MVI), macrovascular invasion, and satellite lesions, tumor differentiation grade, and tumor encapsulation status. Operative variables included operation duration, intraoperative blood loss, the necessity for intraoperative blood transfusion, extent of hepatectomy (minor or major), type of liver resection (anatomical or non-anatomical), resection margin status. Cirrhosis and tumor-related factors were identified through imaging modalities, including ultrasonography, contrast-enhanced computed tomography (CT), and magnetic resonance imaging (MRI) of the abdomen. By definition, major hepatectomy refers to the surgical removal of three or more Couinaud segments via partial hepatectomy, whereas minor hepatectomy involves removing fewer than three segments. Specifically, anatomical liver resection was categorized in accordance with the Brisbane 2000 anatomical classification system, whereas non-anatomical resection comprised wedge resections and limited resections^18^. The serum concentrations of AFP and DCP were determined by the commercially available AR CHITECT immunoassay in accordance with the defined protocol (Abbott Diagnostics). Both AFP and DCP were detected in HCC patients during the week before surgery. AFP positivity was determined as a serum level ≥ 20 ng/ml, and DCP positivity was defined as a serum level ≥ 40 mAU/ml according to the clinically adopted cutoff values.

### Follow-up and primary outcomes

Patients underwent follow-up at each participating hospital, following a tailored surveillance strategy implemented by the institutions. After discharge, patients were monitored using serum AFP or DCP levels, ultrasonography, CT scans, or abdominal MRIs. Surveillance was conducted at intervals of 2-3 months for the first 6 months, 3-4 months for the next 18 months, and subsequently every 3-6 months. Suspected HCC recurrence prompted the performance of clinically indicated tests, including contrast-enhanced CT, MRI, bone scans, or positron emission tomography. Tumor recurrence was defined as the appearance of intrahepatic or extrahepatic tumor nodules, potentially accompanied by an increase in serum AFP or DCP levels. These intrahepatic nodules exhibited imaging features consistent with HCC on CT or MRI scans. Patients with tumor recurrence received active treatments, such as re-resection, transplantation, transcatheter arterial chemoembolization (TACE), percutaneous ablation, radiotherapy, oral sorafenib/lenvatinib, or conservative management, either alone or in combination. Primary study endpoints included survival and recurrence. Overall survival (OS) was measured from the date of liver resection to either the date of death or the last follow-up, while time to recurrence (TTR) was defined as the time from hepatectomy to diagnosis of tumor recurrence or the last follow-up. Recurrence-free survival (RFS) was defined as the time period from the date of surgery to the first occurrence of HCC recurrence or death from any cause.

### Statistical analysis

Continuous variables were reported as either mean ± standard deviation (SD) or median with interquartile range (IQR), depending on whether the data met the normal distribution. Meanwhile, categorical variables were expressed as frequencies and percentages. To account for both survival and recurrence, we set the outcome variable to RFS and the DCP upper limit to 1000 mAU/ml. Based on R package “CatPredi”, the optimal cutoff value for the was determined to be 600 mAU/ml. Baseline characteristics between the two groups (low DCP vs. high DCP) were compared using either a Student's *t* test or a Mann-Whitney U test for continuous variables, and the Chi-square or Fisher's exact tests for categorical variables, based on the suitability of each test. Time-dependent receiver operating characteristic (ROC) curves were employed to assess various cutoff values for DCP discrimination and clinical utility. The area under the ROC curve (AUC) was calculated for each DCP cutoff value, where higher AUC values suggest better discrimination. Using the Kaplan-Meier method examined by the log rank test, OS and TTR rates were compared between the low DCP and high DCP groups. Multivariable Cox regression analyses were performed to determine the independent risk factors related to worse OS and TTR after liver resection. Variable with a *P* < 0.05 in the univariable analysis were included in the multivariable Cox regression model. Subgroup analyses were conducted to assessed the prognostic value serum DCP levels in various groups. Interaction tests were performed to identify whether the impact of preoperative serum DCP levels on OS and TTR differed significantly among the subgroups. Statistical analyses were conducted using R 4.3.1 and SPSS software (version 25.0, SPSS, Inc.). A two-sided *P* < 0.05 was considered statistically significant.

## Results

### Patient characteristics

**Figure 1:**
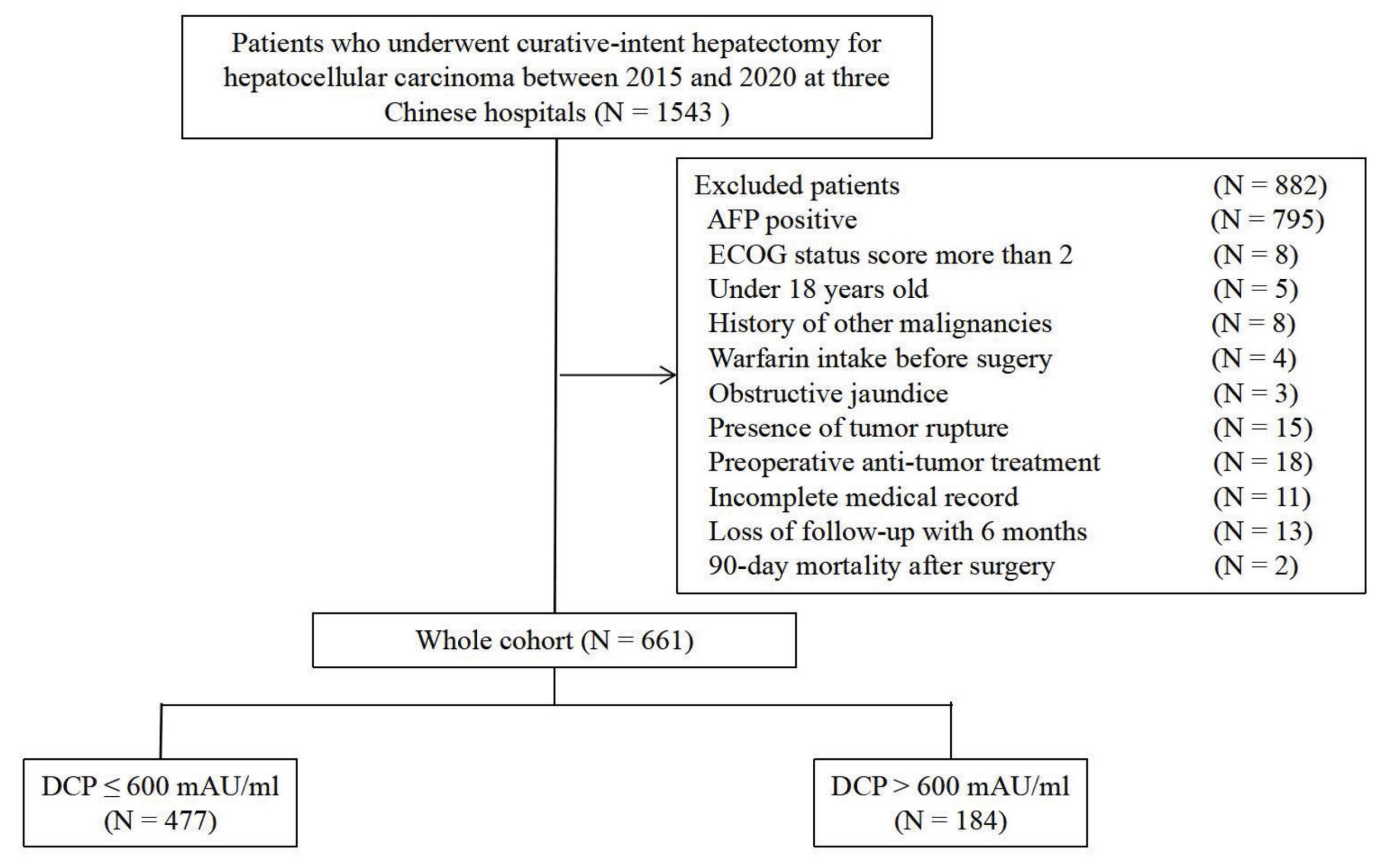
Selection of the study population.

Among 661 patients identified, there were 584 men (88.4%) and 77 women (11.6%) with a median age of 56 (range 23-86) years. Using this cutoff value, patients were divided into low DCP levels (≤ 600 mAU/ml, n = 477) and high DCP levels (> 600 mAU/ml, n = 184) cohorts (***Figure 1***). Baseline characteristics and operative variables differed among patients with and without high DCP level (***Table 1***). Significant differences were observed between the two groups in terms of age, BMI, presence of HBV infection, preoperative AST level, tumor size, tumor number, presence of MVI, macrovascular invasion, and satellites, operation time, intraoperative blood loss, presence of blood transfusion, extent of liver resection, type of hepatectomy (all *P* < 0.05). Patients with high DCP levels were more likely to be elderly, have advanced tumor burden, including tumor size, MVI, macrovascular invasion, and satellites.

### Long-term outcomes

**Figure 2:**
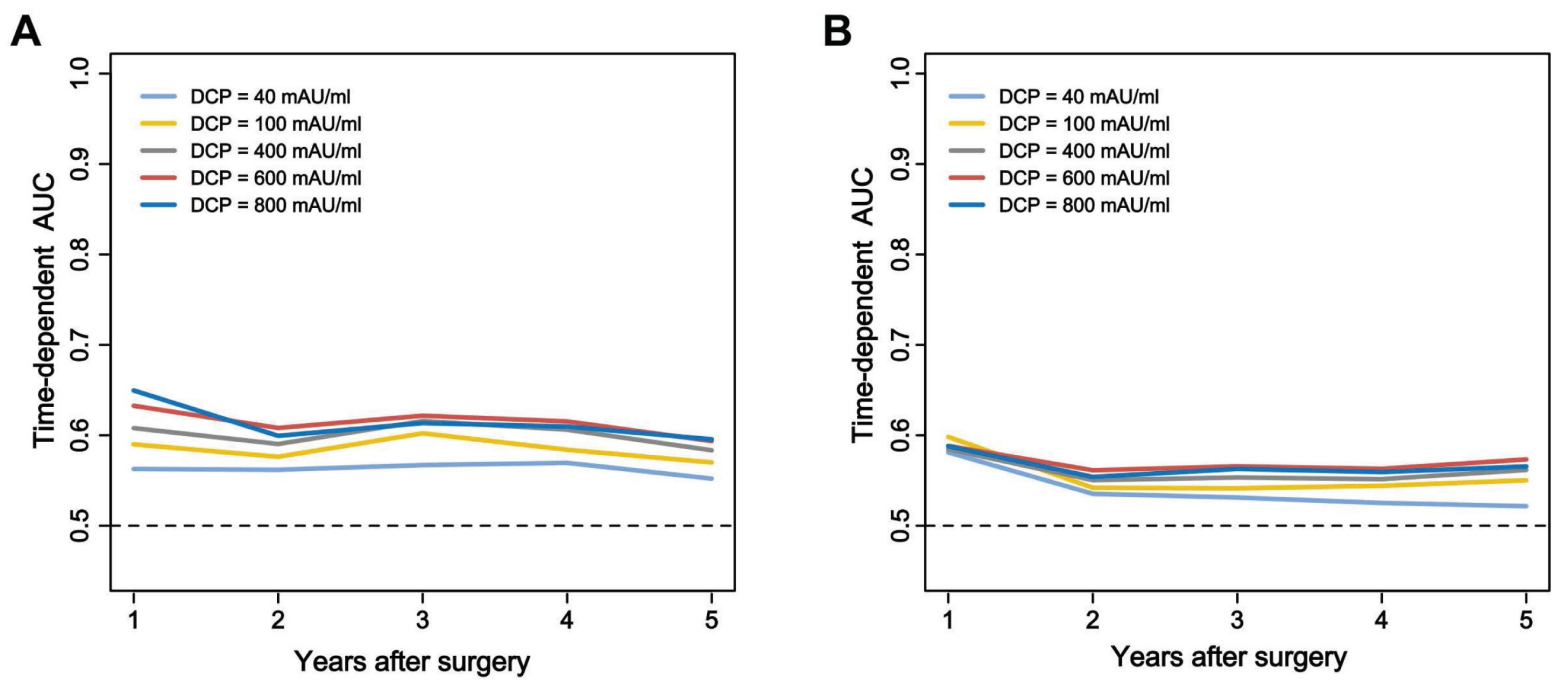
Time-dependent receiver operating characteristic curves comparing the predictive performance of the different cutoff values of DCP for overall survival (**A**) and time to recurrence (**B**).

**Figure 3:**
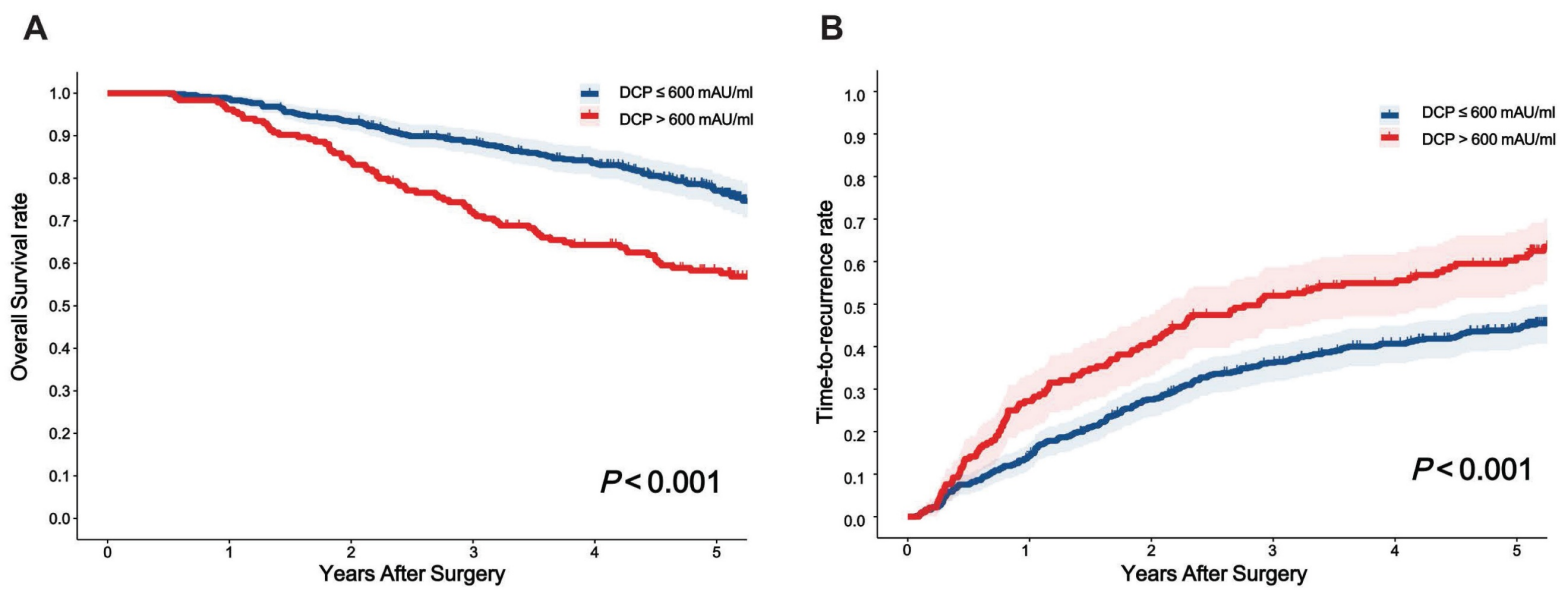
Curves comparisons of overall survival (**A**) and time to recurrence (**B**) between two groups (calculated by log-rank test).

The long-term prognostic outcomes of the 661 patients treated with curative hepatectomy for HCC were stratified according to the DCP levels (***Table 2***). Time-dependent ROC curves analysis confirmed the superior discrimination of 600 mAU/ml as cutoff value for DCP to predict survival and recurrence (***Figure 2***). The mortality rates were 25.6% among patients with low DCP levels and 45.7% in those with high DCP levels (*P* < 0.05). The recurrence rates 45.7% and 61.4% respectively. Compared with low DCP group patients, patients in the high DCP group tended to experience intrahepatic recurrence and the extent of recurrent HCC were more likely to be at an intermediate stage. The 1-, 3-, and 5-year OS and TTR rates among patients with high DCP levels compared to those among patients with low DCP levels are shown in ***Figure 3A*** and ***Figure 3B***.

### Prognostic analyses for overall survival and recurrence

***Table 3*** and ***Table 4*** describe the independent risk factors associated with long-term OS and TTR after curative hepatectomy for HCC confirmed by univariable and multivariable Cox regression analyses. Multivariable analyses demonstrated that high DCP levels were risk factor for decreased OS (HR 1.548, 95%CI 1.135-2.111; *P* = 0.006). Other independent prognostic factors for OS included cirrhosis, tumor size > 5 cm, microvascular invasion, macrovascular invasion, satellites, incomplete tumor encapsulation, narrow resection margin (all *P* < 0.05). Similarly, high DCP levels were risk factor for increased TTR (HR 1.390, 95%CI 1.081-1.787; *P* = 0.010). Other independent prognostic factors for TTR included male, cirrhosis, tumor size > 5 cm, multiple tumors, MVI, macrovascular invasion, satellites, intraoperative blood loss > 400ml, non-anatomical liver resection (all *P* < 0.05).

### Subgroup analyses

**Figure 4:**
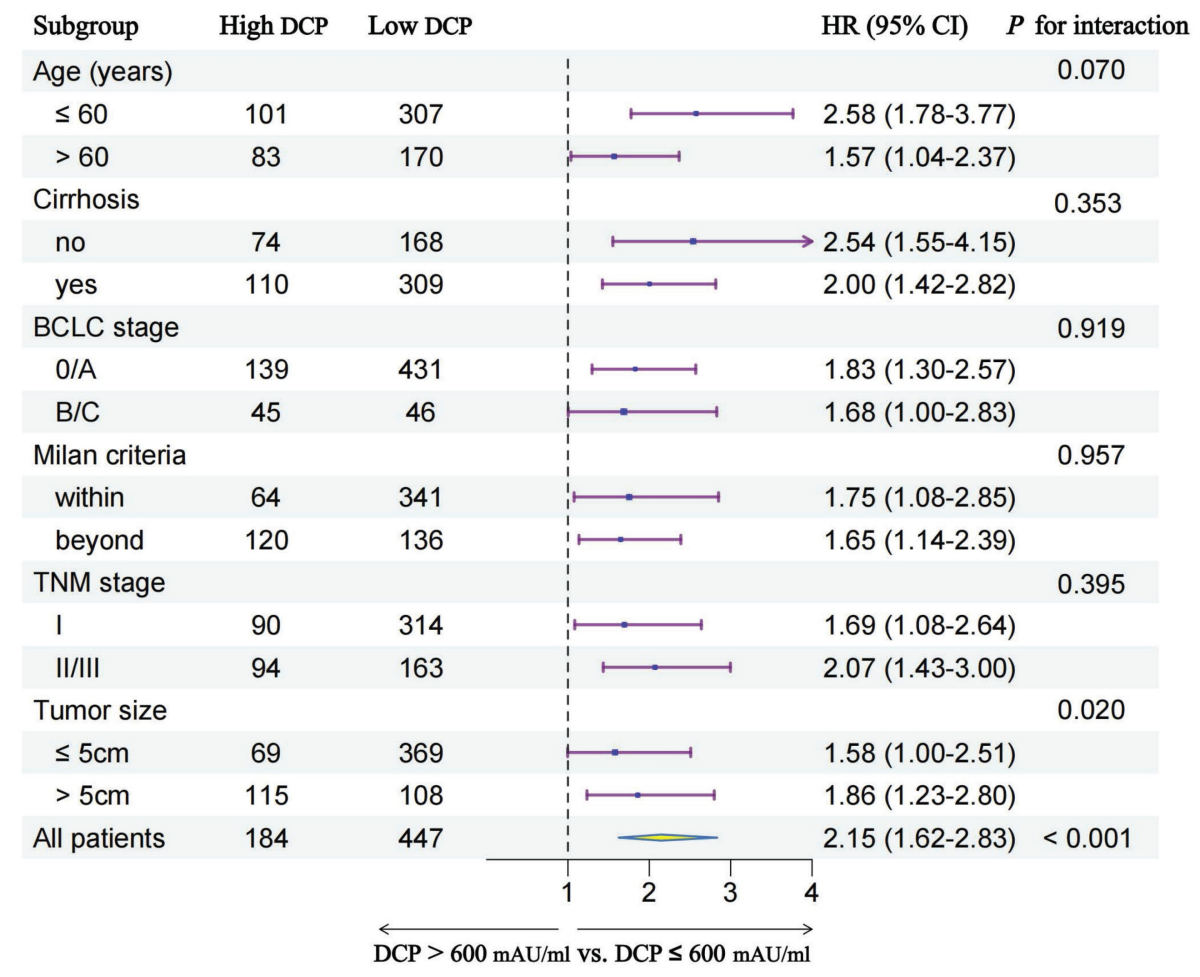
Subgroup analysis of overall survival between two groups.

**Figure 5:**
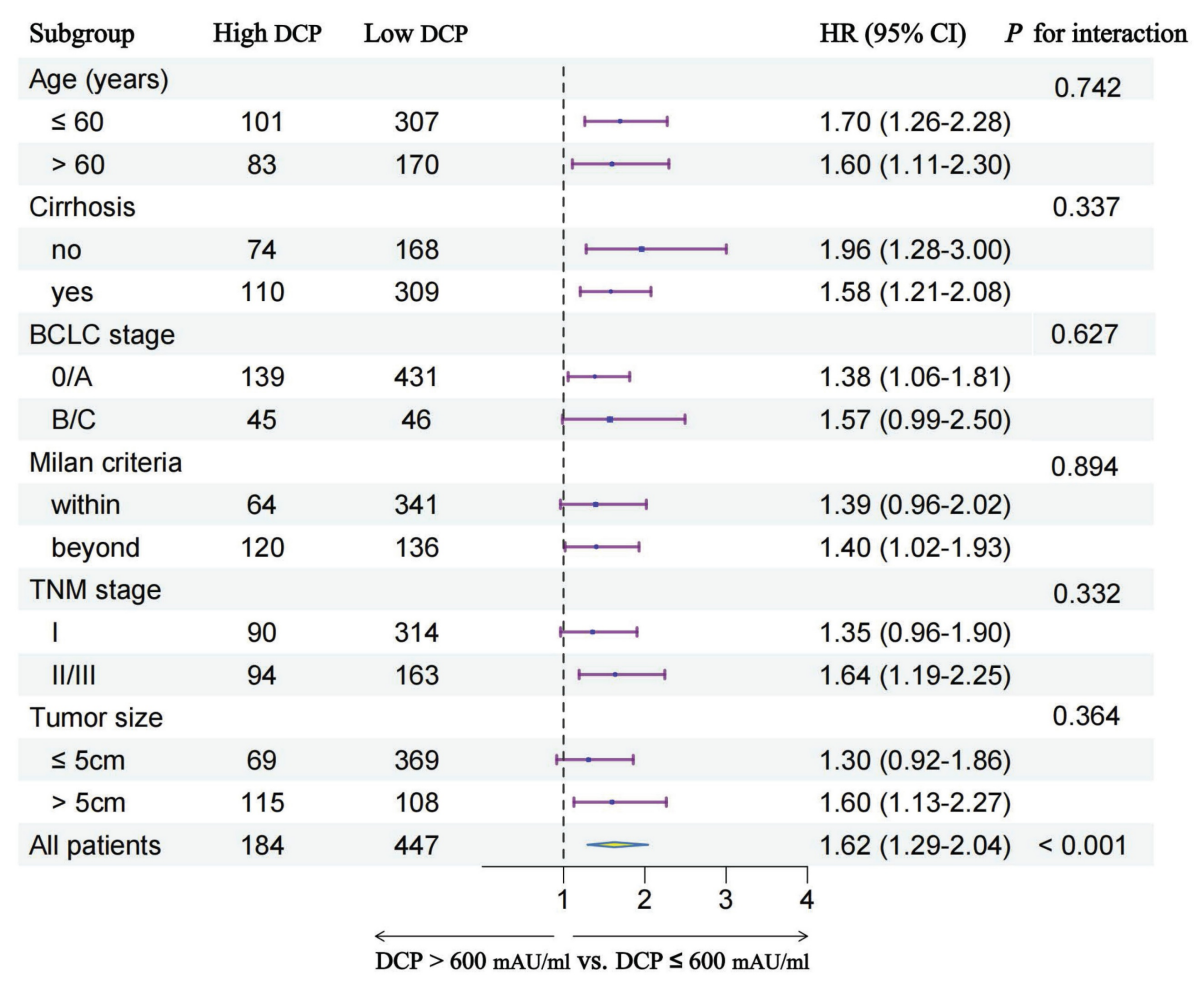
Subgroup analysis of time-to-recurrence between two groups.

In order to further explore the prognostic value of preoperative serum DCP levels in different patient subgroups, we conducted subgroup analyses stratified by multiple clinical characteristics. ***Figure 4*** and ***Figure 5*** show the outcomes of univariable Cox regression analysis for OS and TTR based on the different DCP levels, with high DCP levels linked to poorer OS and TTR in various subgroups, such as age, cirrhosis, tumor size and different tumor staging.

## Discussion

DCP, which was first identified in 1984, not only showed superior sensitivity in the diagnosis of HCC, but also its preoperative levels were associated with the prognosis of HCC patients following liver resection^19^. However, studies on the cutoff values of preoperative DCP as an independent factor for survival and recurrence in patients with HCC were various, especially in liver resection^12^-^14^,^20^-^26^. In order to determine the optimal prognostic cutoff value of preoperative DCP level for HCC patients who underwent liver resection, we selected AFP-negative patients for this study, thus eliminating the combined effect of DCP and AFP on long-term oncologic prognosis. To the best of our knowledge, this is the first study having largest number of HCC patients with AFP-negative after surgery so far regarding DCP. In this large study from a prospectively and retrospectively collected multicenter database, the risk factors, recurrent patterns, and long-term outcomes regarding OS and TTR after curative liver resection for HCC patients with high or low DCP levels (> 600 or ≤ 600 mAU/ml) were evaluated. Among the patients in current study, HBV infection remained the primary etiology, accounting for more than 85%. The results of the cohort of patients undergoing HCC resection revealed that patients with high DCP levels tended to have more aggressive tumor pathological characteristics than the low DCP group patients. Compared with low DCP group patients, patients with high DCP levels were more likely to experience postoperative morbidity.

When we explored the relationship between pathological features and serum DCP level in the cohort, MVI was found in 87 (47.3%) of 184 patients with DCP > 600 mAU/ml, but in only 129 (27.0%) of 477 patients with DCP ≤ 600 mAU/ml, which was similar to the results of previous studies^27^,^28^. Furthermore, HCC patients with high levels of DCP have greater chances of macrovascular invasion and Satellites than those patients with lower levels of DCP^29^. As far as long-term oncologic prognosis is concerned, patients in the high DCP group had worse 5-year survival rate (57.6% vs. 76.3%, *P* < 0.001) and 5-year recurrence rate (61.0 vs. 44.9%, *P* < 0.001) than those in the low DCP group. Meanwhile, multivariable Cox regression identified that high DCP levels was an independent risk factor for decreased OS and elevated TTR, a finding also consistently reported in other studies^20^,^30^. In addition to some confirmed factors, such as cirrhosis, tumor size, tumor number, vascular invasion, tumor encapsulation, and resection margin, are associated with long-term prognosis for HCC. Interestingly, we observed that male were an independent risk factor for postoperative recurrence in AFP-negative HCC patients, which may be due to differences in sex hormones^31^,^32^ and the greater propensity of men to drink alcohol, leading to more severe cirrhosis^33^,^34^. Furthermore, patients with elevated DCP levels were at an increased risk for intrahepatic recurrence^21^,^35^, often presenting at BCLC B/C stages, potentially owing to the high occurrence of MVI. Several traditional HCC staging systems based on tumor burden and vascular invasion are widely used to guide clinical decision-making, such as the BCLC stage^6^, AJCC TNM stage^7^, and the Milan criteria^36^. However, these tumor stagings did not take into account indicators reflecting tumor biological characteristics. In this study, we further stratified AFP-negative HCC patients after surgery according to preoperative DCP levels in different subgroup. In general, the prognostic of preoperative DCP, as a HCC-specific tumor marker, was consistent across the different tumor stagings, which proved that different DCP levels are a good supplement for clinical tumor staging.

Apart from indicating poor long-term prognosis of patients, positive preoperative DCP levels can be identified by timely postoperative follow-up. Dynamic observation of DCP changes to evaluate the efficacy of therapeutic interventions and the surveillance of recurrences^37^. For patients with unresectable HCC, the use of DCP as a prognostic marker exhibits dual characteristics. The primary treatment modalities for these patients include interventional therapy and molecular targeted drug therapy. Therefore, in some cases, DCP levels may increase in response to anti-VEGF therapy and TACE^38^,^39^. This elevation is attributed to the hypoxic environment induced by anti-VEGF agents and local arterial embolization within the tumor, which results in vitamin K deficiency. This means that elevated levels of DCP may indicate two possibilities: either that systemic therapy is effective or that it is ineffective and the tumor is progressing. For HCC patients who underwent hepatectomy with positive preoperative levels of DCP, when the first postoperative follow-up levels did not decrease to normal or to a specific percentage due to extremely high preoperative DCP levels, we need to be alert to the possibility that residual microlesions or circulating tumor cells may exist despite the removal of the neoplasm and surgeons should consider aggressive postoperative adjuvant treatment options^1^,^40^.

Our study has some limitations. First, the study was retrospective in nature, which could lead to inherent biases. In addition, chronic HBV infection, accounting for 85.6% of the whole cohort, is the primary cause of HCC in this study, and differs from the pattern of disease observed in the United States and other Western countries. Furthermore, The variability in test results for DCP across different hospitals poses challenges for conducting large-scale studies. Last, This study focused on AFP-negative HCC patients, and further research is needed in the general patients.

## Conclusion

A DCP cutoff value of 600 mAU/ml effectively stratifies AFP-negative HCC patients into high- and low-risk groups and high DCP level is an independent risk factor for OS and TTR after liver resection. Both preoperative DCP value and changes when postoperative follow-up can guide clinical decision-making.

## Figures and Tables

**Table 1:** Baseline characteristics of the patients in the two cohorts.

N (%)	Total (N = 661)	DCP ≤ 600 mAU/ml (N = 477)	DCP > 600 mAU/ml (N = 184)	*P* value
Clinicalpathologic Characteristics				
Age > 60 years	253 (38.3)	170 (35.6)	83 (45.1)	0.025
Male	584 (88.4)	425 (89.1)	159 (86.4)	0.335
ASA score > 2	115 (17.4)	81 (17.0)	34 (18.5)	0.649
Body mass index > 24 Kg/m^2^	350 (53.0)	269 (56.4)	81 (44.0)	0.004
HBV (+)	566 (85.6)	419 (87.8)	147 (79.9)	0.009
HCV (+)	16 (2.4)	10 (2.1)	6 (3.3)	0.555
Cirrhosis	419 (63.4)	309 (64.8)	110 (59.8)	0.232
Portal hypertension	99 (15.0)	70 (14.7)	29 (15.8)	0.726
Child-Pugh grade B	25 (3.8)	21 (4.4)	4 (2.2)	0.178
Preoperative ALT level > 40 U/L	147 (22.2)	102 (21.4)	45 (24.5)	0.395
Preoperative AST level > 40 U/L	101 (15.3)	57 (11.9)	44 (23.9)	<0.001
Largest tumor diameter > 5 cm	223 (33.7)	108 (22.6)	115 (62.5)	<0.001
Multiple Tumors	74 (11.2)	61 (12.8)	13 (7.1)	0.036
Microvascular invasion	216 (32.7)	129 (27.0)	87 (47.3)	<0.001
Macrovascular invasion	9 (1.4)	3 (0.6)	6 (3.3)	0.025
Satellites	38 (5.7)	22 (4.6)	16 (8.7)	0.043
Poor tumor differentiation	483 (73.1)	351 (73.6)	132 (71.7)	0.632
Incomplete tumor encapsulation	456 (69.0)	335 (70.2)	121 (65.8)	0.265
Operative Variables				
Operation time	125 (100-158)	120 (95-150)	145 (120-180)	<0.001
Intraoperative blood loss > 400 ml	53 (8.0)	28 (5.9)	25 (13.6)	0.001
Intraoperative blood transfusion	34 (5.1)	16 (3.4)	18 (9.8)	0.001
Major liver resection	82 (12.4)	39 (8.2)	43 (23.4)	<0.001
Non-anatomical liver resection	577 (87.3)	429 (89.9)	148 (80.4)	0.001
Resection margin < 1cm	416 (62.9)	291 (61.0)	125 (67.9)	0.098
Perioperative Outcomes				
Postoperative 30-day morbidity	195 (29.5)	130 (27.3)	65 (35.3)	0.041
Minor morbidity	165 (25.0)	110 (23.1)	55 (29.9)	0.069
Major morbidity	30 (4.5)	20 (4.2)	10 (5.4)	0.414
Postoperative hospital stays, days	8.3±3.7	8.4±4.1	8.2±2.3	0.687

**Abbreviations:** DCP, des-gamma-carboxy prothrombin; ASA, American Society of Anesthesiologists; HBV, hepatitis B virus; HCV, hepatitis C virus; AST, aspartate transaminase; ALT, alanine aminotransferase.

**Table 2:** Long-term outcomes after excluding postoperative early deaths.

N (%)	Total (N = 661)	DCP ≤ 600 mAU/ml (N = 477)	DCP > 600 mAU/ml (N = 184)	*P* value
Period of follow-up, months	56.5±20.3	58.9±19.4	50.4±21.5	<0.001
Recurrence during the follow-up	331 (50.1)	218 (45.7)	113 (61.4)	<0.001
Intrahepatic recurrence	310 (46.9)	204 (42.8)	106 (57.6)	0.001
Extrahepatic recurrence	4 (0.6)	4 (0.8)	0 (0.0)	0.580
Intra- & extrahepatic recurrence	17 (2.6)	10 (2.1)	7 (3.8)	0.217
BCLC stage of initial recurrence	331 (50.1)	218 (45.7)	113 (61.4)	<0.001
BCLC A stage	216 (32.7)	153 (32.1)	63 (34.2)	0.595
BCLC B stage	84 (12.7)	47 (9.9)	37 (20.1)	<0.001
BCLC C stage	31 (4.7)	18 (3.8)	13 (7.1)	0.073
Mortality during the follow-up	206 (31.2)	122 (25.6)	84 (45.7)	<0.001
Median overall survival (OS), 95% CI	61.5 (44.9-68.4)	61.7 (51.7-69.7)	58.5 (32.5-65.7)	<0.001
1-year OS rate, %	97.9%	98.5%	95.1%	
3-year OS rate, %	83.4%	88.2%	71.1%	
5-year OS rate, %	71.1%	76.3%	57.6%	
Median time-to-recurrence (TTR), 95% CI	46.3 (17.3-63.9)	54.0 (20.6-65.1)	58.5 (10.5-61.4)	<0.001
1-year TTR rate, %	18.0%	16.8%	28.8%	
3-year TTR rate, %	40.6%	36.2%	52.0%	
5-year TTR rate, %	49.3%	44.9%	61.0%	

**Table 3:** Univariable and multivariable Cox regression analyses of risk factors for survival.

Variables	HR comparison	UV HR (95% CI)	UV *P*	MV HR (95% CI)	MV *P*
Age	> 60 *vs.* ≤ 60 years	1.397 (1.062-1.839)	0.017	NA	0.062
Sex	Male *vs.* Female	0.890 (0.594-1.335)	0.574		
ASA score	> 2 *vs.* ≤ 2	1.269 (0.904-1.781)	0.169		
Body mass index	> 24 *vs.* ≤ 24 Kg/m^2^	0.739 (0.562-0.972)	0.030	NA	0.053
HBV (+)	Negative *vs.* Negative	0.752 (0.525-1.078)	0.121		
Cirrhosis	Presence *vs.* Absence	1.334 (0.993-1.792)	0.056	1.362 (1.008-1.842)	0.045
Portal hypertension	Presence *vs.* Absence	0.992 (0.677-1.454)	0.968		
Child-Pugh grade	B *vs.* A	1.416 (0.771-2.601)	0.262		
Preoperative ALT level	> 40* vs.* ≤ 40 U/L	1.171 (0.854-1.607)	0.327		
Preoperative AST level	> 40* vs.* ≤ 40 U/L	1.595 (1.140-2.233)	0.006	NA	0.564
Preoperative DCP level	> 600 *vs.* ≤ 600 mAU/ml	2.146 (1.624-2.835)	< 0.001	1.548 (1.135-2.111)	0.006
Largest tumor diameter	> 5 *vs.* ≤ 5 cm	2.077 (1.580-2.731)	< 0.001	1.811 (1.324-2.477)	< 0.001
Tumor number	Multiple *vs.* Solitary	1.323 (0.893-1.959)	0.163		
Microvascular invasion	Yes *vs.* no	2.452 (1.864-3.225)	< 0.001	2.096 (1.574-2.792)	< 0.001
Macrovascular invasion	Yes *vs.* no	7.314 (3.585-14.920)	< 0.001	2.498 (1.189-5.251)	0.016
Satellites	Yes *vs.* no	3.504 (2.305-5.327)	< 0.001	2.048 (1.317-3.187)	0.001
Poor tumor differentiation	Yes *vs.* no	1.069 (0.783-1.458)	0.675		
Incomplete tumor encapsulation	Yes *vs.* no	1.573 (1.145-2.160)	0.005	1.757 (1.269-2.431)	0.001
Intraoperative blood loss	> 400* vs.* ≤ 400 ml	1.969 (1.295-2.993)	0.002	NA	0.081
Intraoperative transfusion	Yes *vs.* no	2.033 (1.237-3.342)	0.005	NA	0.190
Extent of liver resection	Major* vs.* ≤ Minor	1.726 (1.199-2.484)	0.003	NA	0.631
Non-anatomical liver resection	Yes *vs.* no	0.935 (0.624-1.401)	0.743		
Resection margin	< 1 *vs.* ≥ 1 cm	1.888 (1.375-2.592)	< 0.001	1.665 (1.207-2.297)	0.002

**Abbreviations:** DCP, des-gamma-carboxy prothrombin; ASA, American Society of Anesthesiologists; HBV, hepatitis B virus; HCV, hepatitis C virus; AST, aspartate transaminase; ALT, alanine aminotransferase; CI, Confidence interval; HR, Hazard ratio; MV, multivariable; UV, univariable.

**Table 4:** Univariable and multivariable Cox regression analyses of risk factors for recurrence.

Variables	HR comparison	UV HR (95% CI)	UV *P*	MV HR (95% CI)	MV *P*
Age	> 60 *vs.* ≤ 60 years	0.935 (0.748-1.169)	0.556		
Sex	Male *vs.* Female	1.419 (0.975-2.066)	0.067	1.645 (1.123-2.409)	0.011
ASA score	> 2 *vs.* ≤ 2	0.771 (0.569-1.045)	0.094	NA	0.073
Body mass index	> 24 *vs.* ≤ 24 Kg/m^2^	0.934 (0.752-1.159)	0.535		
HBV (+)	Negative *vs.* Negative	1.363 (0.968-1.918)	0.076	NA	0.120
Cirrhosis	Presence *vs.* Absence	1.811 (1.418-2.313)	< 0.001	1.876 (1.462-2.407)	< 0.001
Portal hypertension	Presence *vs.* Absence	1.123 (0.841-1.501)	0.431		
Child-Pugh grade	B *vs.* A	1.289 (0.780-2.132)	0.322		
Preoperative ALT level	> 40* vs.* ≤ 40 U/L	1.271 (0.992-1.629)	0.057	NA	0.325
Preoperative AST level	> 40* vs.* ≤ 40 U/L	1.394 (1.054-1.844)	0.020	NA	0.414
Preoperative DCP level	> 600 *vs.* ≤ 600 mAU/ml	1.625 (1.294-2.041)	< 0.001	1.390 (1.081-1.787)	0.010
Largest tumor diameter	> 5 *vs.* ≤ 5 cm	1.545 (1.239-1.927)	< 0.001	1.550 (1.200-2.002)	0.001
Tumor number	Multiple *vs.* Solitary	1.602 (1.177-2.180)	0.003	1.552 (1.134-2.124)	0.006
Microvascular invasion	Yes *vs.* no	1.612 (1.291-2.013)	< 0.001	1.439 (1.141-1.815)	0.002
Macrovascular invasion	Yes *vs.* no	4.428 (2.190-8.954)	< 0.001	2.823 (1.356-5.876)	0.006
Satellites	Yes *vs.* no	3.165 (2.182-4.590)	< 0.001	2.089 (1.406-3.103)	< 0.001
Poor tumor differentiation	Yes *vs.* no	1.193 (0.930-1.529)	0.165		
Incomplete tumor encapsulation	Yes *vs.* no	1.251 (0.985-1.589)	0.066	NA	0.051
Intraoperative blood loss	> 400* vs.* ≤ 400 ml	1.726 (1.215-2.451)	0.002	1.615 (1.125-2.320)	0.009
Intraoperative transfusion	Yes *vs.* no	1.077 (0.652-1.781)	0.771		
Extent of liver resection	Major* vs.* ≤ Minor	1.225 (0.889-1.688)	0.215		
Non-anatomical liver resection	Yes *vs.* no	1.406 (0.981-2.014)	0.064	1.679 (1.156-2.347)	0.006
Resection margin	< 1 *vs.* ≥ 1 cm	1.551 (1.227-1.962)	< 0.001	NA	0.088

**Abbreviations:** DCP, des-gamma-carboxy prothrombin; ASA, American Society of Anesthesiologists; HBV, hepatitis B virus; HCV, hepatitis C virus; AST, aspartate transaminase; ALT, alanine aminotransferase; CI, Confidence interval; HR, Hazard ratio; MV, multivariable; UV, univariable.
